# The control of sea lice in Atlantic salmon by selective breeding

**DOI:** 10.1098/rsif.2015.0574

**Published:** 2015-09-06

**Authors:** Karim Gharbi, Louise Matthews, James Bron, Ron Roberts, Alan Tinch, Michael Stear

**Affiliations:** 1 Institute of Biodiversity, Animal Health and Comparative Medicine, University of Glasgow, Glasgow G61 1QH, UK; 2 Boyd Orr Centre for Population and Ecosystem Health, University of Glasgow, Glasgow, UK; 3 Institute of Aquaculture, University of Stirling, Stirling FK9 4LA, UK; 4 Landcatch Natural Selection Ltd, Alloa FK10 3LP, UK

**Keywords:** sustainable agriculture, Atlantic salmon, sea lice, *Lepeophtheirus salmonis*, selective breeding, epidemiological modelling

## Abstract

Sea lice threaten the welfare of farmed Atlantic salmon and the sustainability of fish farming across the world. Chemical treatments are the major method of control but drug resistance means that alternatives are urgently needed. Selective breeding can be a cheap and effective alternative. Here, we combine experimental trials and diagnostics to provide a practical protocol for quantifying resistance to sea lice. We then combined quantitative genetics with epidemiological modelling to make the first prediction of the response to selection, quantified in terms of reduced need for chemical treatments. We infected over 1400 young fish with *Lepeophtheirus salmonis*, the most important species in the Northern Hemisphere. Mechanisms of resistance were expressed early in infection. Consequently, the number of lice per fish and the ranking of families were very similar at 7 and 17 days post infection, providing a stable window for assessing susceptibility to infection. The heritability of lice numbers within this time window was moderately high at 0.3, confirming that selective breeding is viable. We combined an epidemiological model of sea lice infection and control on a salmon farm with genetic variation in susceptibility among individuals. We simulated 10 generations of selective breeding and examined the frequency of treatments needed to control infection. Our model predicted that substantially fewer chemical treatments are needed to control lice outbreaks in selected populations and chemical treatment could be unnecessary after 10 generations of selection. Selective breeding for sea lice resistance should reduce the impact of sea lice on fish health and thus substantially improve the sustainability of Atlantic salmon production.

## 1. Introduction

Infection of Atlantic salmon by the salmon louse, *Lepeophtheirus salmonis*, is a major threat to fish welfare and the profitability of salmon production. *L. salmonis* can cause skin lesions, osmotic imbalance, and increased susceptibility to bacterial and viral infections through suppression of host immune responses and damage to the host skin [1]. Salmon farms combat sea lice with chemical treatments. Treatment costs vary among countries but amounted to losses of $480 M per annum worldwide in 2006 [2]. However, this figure does not include indirect losses due to fish stress and reduced growth, the potential role of lice as vectors in the transmission of fish pathogens such as infectious salmon anaemia virus [3], the importance of louse infections in increasing susceptibility to other diseases, the environmental impact of chemical treatments [4–7] and the potential impacts on wild salmon stocks [8–13].

Chemical treatments are currently the major control method but increasing concern about the development of resistance by sea lice [14–16] means alternative controls are needed [17,18] and the importance of integrated pest management plans is being recognized [19,20]. Breeding for resistance is now recognized as a key element in the management of disease in intensive animal production [21–23]. There are examples of successful breeding programmes for disease resistance in the salmon aquaculture industry (e.g. those targeting infectious pancreatic necrosis), but, despite estimates of heritabilities for sea lice infection [24–26], programmes to develop commercial stocks showing resistance to sea lice have lacked two key elements. First, they lack reliable, practical protocols for estimating breeding values—the contribution to the lice count from inherited genetic variation. Second, they lack predictions of the response to selection. For production traits, such as growth or milk yield, the response to selective breeding is a straightforward calculation [27]. For infectious disease traits, however, selective breeding alters transmission dynamics and therefore alters an individual's environment as well as its genetic susceptibility. In this case, predicting the response to selection requires quantitative genetics to be integrated into epidemiological models.

In this study, we established a standardized challenge for measuring salmon resistance to sea lice infection, estimated the heritability of resistance to sea lice in young fish and developed a mathematical model to predict the response to selection for increased resistance to sea lice. Specifically, we estimated the heritability of abundance of lice during the early and late stages of initial infection. These estimates enabled us to identify the time window during the infection process that maximizes differences between families, while minimizing fish stress. We also compared whole-body counts with individual side counts to help design accurate yet practical industry protocols. Our mathematical model of parasite transmission dynamics accounted for genetic variation in fish resistance and allowed us to compare the effect of selection under alternative management scenarios. The model predicts the expected parasite loads in both selected and unselected individuals and estimates the potential for reduction in the frequency of chemical treatments in selected populations.

## 2. Material and methods

### 2.1. Experimental infection trials

**Figure 1.**
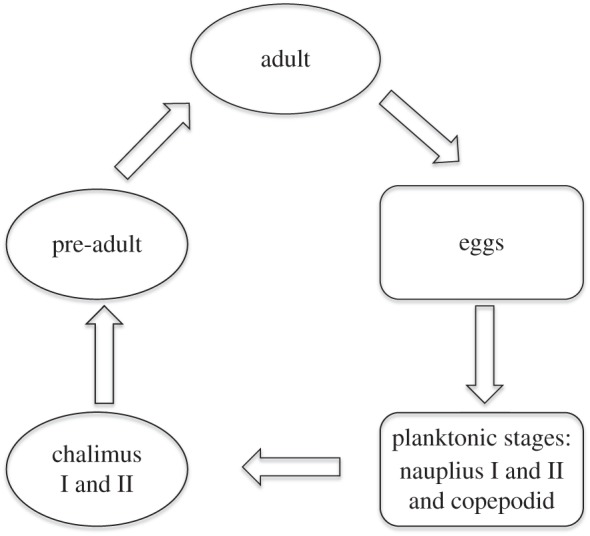
A simplified diagram of the life cycle of sea lice. Following hatching, the sea louse has eight stages to its life cycle [28]. There are three planktonic stages, nauplius I and II that moult into the infective copepodid stage which attaches to the fish. The copepodid stage moults to the sessile stages of the life cycle, chalimus I and II, before becoming the pre-adult or mobile stage that can move around on the surface of the fish and also swim in the water column. The pre-adult stage is followed by the final moult to the fully mature adult stage.

Sea lice have a direct life cycle comprising eight stages separated by moults [28]. The key infectious stage is the free-swimming copepodid that establishes contact with the host following a short planktonic phase (figure 1).

The infection trial was carried out at the Marine Environmental Research Laboratory at Machrihanish. Salmon smolts, at 1 year after hatching, were purchased from Landcatch Natural Selection Ltd (*N* = 1479). The salmon came from 31 sire families with an average of 45 fish per family. Salmon were challenged in a single tank with a moderate dose of copepodid larvae (96 per fish) and monitored daily until the majority of parasites had moulted into chalimus I (figure 1). Seven days post infection (dpi) approximately half of the fish (*N* = 725) were sampled over a 10 h period following euthanasia with benzocaine. Each fish was identified using a passive integrated transponder (PIT) tag, weighed, measured and fixed in 10% neutral buffered formalin, with a fin clip separately archived in ethanol. The remainder of the fish was monitored until most of the lice had reached the chalimus II stage (17 dpi) and was sampled as above. All lice for each fish were counted using a stereo-microscope (Olympus SZ-40). Days 7 and 17 were chosen to cover the developmental stages of the parasite. These timings will vary with water temperature. Day 7 is close to the start of infection but allows lice to be seen and counted more easily. Day 17 is just before moulting into motile pre-adults. Although pre-adults and adults are easier to count than developmental stages their ability to move between hosts means these counts would not reflect the resistance status of the host.

The experiment was designed to allow the estimation of the heritability of susceptibility to sea lice infection. Heritability is defined as the proportion of the total variation that is due to inherited genetic variation [29] and is estimated from the resemblance among relatives [30]. Statistical analysis of louse counts was carried out using SAS v. 9.3 (SAS Institute Cary, NC, USA), and R v. 3.2.0 [31]. Generalized linear mixed modelling with a negative binomial error structure and dpi as a fixed effect as well as sire and dam as random effects was used to compare counts at 7 and 17 dpi. Pedigree information and louse counts were analysed using the ASReml program [32] to generate heritability values in an animal model [30,33]. Lice counts were log transformed for the heritability analysis.

### 2.2. Epidemiological model with selective breeding and sea lice control

The epidemiological model of sea lice infection and control was based on a previously published model which described the epidemiological dynamics of sea lice infection in salmon farms in Scotland over the 2 year production cycle and simulated the effect of treatment with hydrogen peroxide and cypermethrin [34]. This model did not account for heterogeneity in susceptibility between individual fish.

Adopting the notation of Revie *et al*. [34], the rate of arrival of infective stages per fish at time *t* is given by2.1
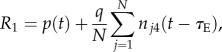
where *p*(*t*) is the background infection pressure, *q* is the number of eggs per female per day, *N* is the number of fish, *n_j_*_4_ is the number of gravid females on fish *j* and *τ*_E_ is the time taken for an egg to develop to the chalimus stage. The background infection pressure takes the form of a Heaviside function2.2
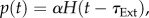
where *α* is the background level of infection and *τ*_Ext_ is the time at which external infection occurs. The dynamics of the sea lice stages on the fish are given by the delay differential equations2.3
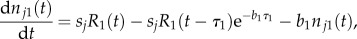
2.4
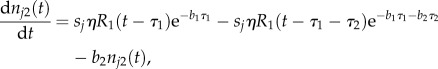
2.5
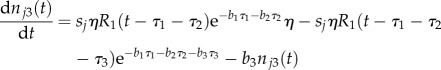
and2.6
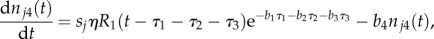
where *n_j_*_1_ is the number of chalimus on fish *j* and *n_j_*_2_, *n_j_*_3_ and *n_j_*_4_ are, respectively, the number of pre-adult, adult and gravid females on fish *j*. The parameter *η* gives the proportion of chalimus developing into females (i.e. *η* = 0.5), and the parameter *s_j_* is the relative susceptibility of fish *j* to sea lice infection. This parameter distinguishes our model from the original model of Revie *et al*. [34], which modelled a single fish or equivalently a population of identical fish, i.e. *s_j_* = 1 for all fish. By assigning individual susceptibilities *s_j_* to each fish, we extend the original model to capture individual variation, both genetic and non-genetic, in resistance to infection. See the next section for a discussion of the genetic theory incorporated into the modelling.

The parameters *b*_1_,*b*_2_,*b*_3_ and *b*_4_ are, respectively, the death rates in the chalimus, pre-adult, adult and gravid stages, and the parameters *τ*_1_,*τ*_2_,*τ*_3_,*τ*_4_, are the expected number of days spent in the respective stages.

Following Revie *et al*. [34], the effect of treatment was simulated by assuming a reduction in the population on each fish and the chalimus source term, given by2.7
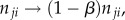


The parameters taken from Revie *et al*. [34] are given in table 1.

**Table 1.** Parameters for the epidemiological component of the sea lice model taken from Revie *et al*. [34]. The parameters *β*, *S_C_*, *S_C_*, *α* and *q* are specific to farms treating with cypermethrin.

parameter	value	description
*τ*_1_	15	days spent in stage 1
*τ*_2_	20	days spent in stage 2
*τ*_3_	10	days spent in stage 3
*τ*_4_	12	days spent in stage 4
*τ*_E_	20	egg to chalimus development time (days)
*τ*_Ext_	154	external infection arrival day
q	8.745	viable eggs per female per day
*α*	1.025	background chalimus per day
*_*β*_*	0.95	treatment efficacy
*S_C_*	0.642	survival fraction to next stage
*S_M_*	0.973	survival fraction to next stage
*b*_1_ = −ln(*S_C_*)/*τ*_1_	0.030	mortality rate of stage 1
*b*_2_ = −ln(*S_M_*)/*τ*_2_	0.0014	mortality rate of stage 2
*b*_3_ = −ln(*S_M_*)/*τ*_3_	0.0027	mortality rate of stage 3
*b*_4_ = −ln(2)/*τ*_4_	0.058	mortality rate of stage 4

### 2.3. Genetic variation in the epidemiological model

The distribution of susceptibilities *s_j_* and the heritability was, *h*^2^, based on our lice count data from our experimental trials. Specifically, the pooled distributions of lice counts (log transformed) were used to define the variation in susceptibility to infection, and the pooled heritability estimate used to specify the extent of inherited variation in susceptibility.

**Figure 2.**
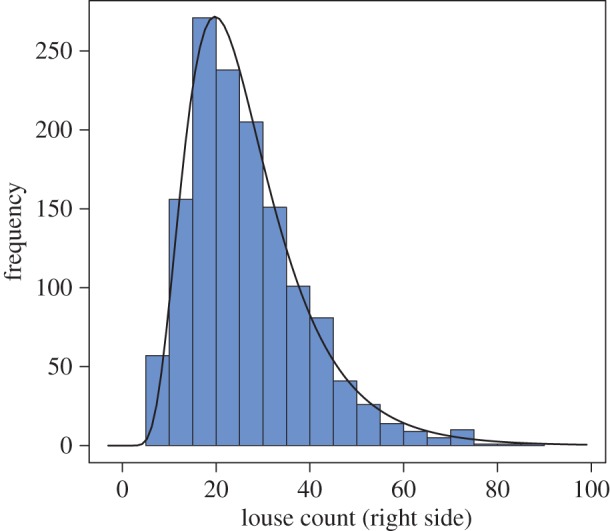
Frequency distribution of number of lice counted from right side of salmon only (blue bars) with corresponding lognormal distribution overlaid (line). The distribution of lice counts is right skewed. Most fish have relatively low counts but a small number of fish have high counts. A total of 1405 fish were examined.

As the lice counts were lognormally distributed (figure 2), we assumed susceptibility to follow the equivalent lognormal distribution rescaled to a mean of 1.0. As standard quantitative genetic theory [27] applies to normally distributed traits, this underlying normal distribution (the log of susceptibility) was assumed to be the trait subject to selection.

Specifically, prior to selection the trait was assumed to follow a normal distribution with density function *ϕ*((*x* − *μ*)/*σ*)/*σ*, with mean, *μ*, standard deviation, *σ*, where *ϕ*(*x*) is the standard normal distribution density function given by2.8
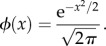


We simulated 10 generations of selection, assuming truncated selection, which assumes a proportion, *p*, of the population is selected for breeding, corresponding here to selection of individuals below a threshold value for the trait, *T*.

The response to selection, *R*, is the difference in mean phenotypic value between the parental generation and the offspring, and depends on the heritability, *h*^2^, of the trait. The standard expression for the response to selection, *R*, is given by the breeder's equation [27]2.9
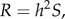
where *S* is the selection differential, *S*, which is equal to the average superiority of the selected parents, i.e.2.10
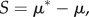
where μ* is the mean of the selected population. In this case, *S* will be negative because we are selecting individuals with the lowest lice counts. This may also be written2.11
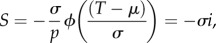
where2.12
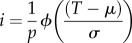
is the intensity of selection, a standardized measure of the proportion of the population being used for selection [27].

The breeder's equation (equation (2.9)) assumes no change in variance due to selection, reflecting the infinitesimal model, which assumes a very large (effectively infinite) number of loci each with infinitesimal effect. Under this model, the amount of selection acting at any given locus is small and therefore that the change in allele frequencies is negligible.

However, short-term changes in variance are assumed to occur via the Bulmer effect [27,35]. The Bulmer effect captures the reduction in variance due to the disequilibrium among loci that arises in a selected population; it is short term because random mating is assumed to rapidly restore equilibrium.

Specifically, truncated selection reduces the trait variance in the selected parental population [27] to2.13
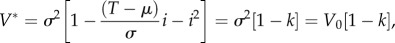
where2.14
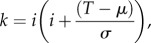
where *V*_0_ is the phenotypic variance prior to selection. Note that this expression (2.14) differs slightly from standard because we are selecting individuals with the lowest values for the trait, rather than the highest. Following the notation of [35], this decrease in variance in the parental generation reduces the variance in the first offspring generation by
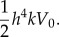
This is a temporary reduction generated by linkage disequilibrium. We use *d_i_* to denote the disequilibrium contribution at the *i*th generation. In each generation of selection, the existing disequilibrium contribution is halved and a new contribution generated [27,35], i.e.
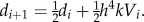


Denoting the pre-selection additive and phenotypic variances by *A*_0_ and *V*_0_, respectively, the additive and phenotypic variances and the heritability in the *i*th generation are given
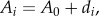

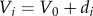
and
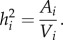


In our simulated selection schemes, we account in each generation for this reduction in variance and heritability in the response to selection.

### 2.4. Simulation studies

We used the epidemiological model to simulate infection dynamics and control first in the absence of selective breeding and then on selected populations. We predicted the response to selection in terms of reduction in mean parasite load across the fish population and the required frequency of treatment to maintain lice below a threshold over the 2 year production cycle, following Revie *et al*. [34].

From the breeder's equation, the key parameters affecting the response to selection are the heritability, the intensity of selection and the variance in the trait prior to selection. Though changes to the epidemiological parameters would affect absolute lice numbers, they would have little impact on the relative reduction in population growth and treatment frequencies, which are our focus. We ran simulations for a wide range of selection intensities from selection on the best 80% of the population down to selection on just 1% of population. In aquaculture, the large numbers of offspring mean that extreme selection intensities down to 1% of the population are feasible. We also investigated the sensitivity of the response to selection (in terms of reduction in treatment frequency) to the heritability and the variance in susceptibility prior to selection.

## 3. Results

### 3.1. Experimental infection trials

Lice counts were obtained for a total of 1405 fish. To determine when mechanisms of resistance are expressed, 691 and 714 fish were sampled at the early (7 dpi) and late (17 dpi) infection stages, respectively. To establish a practical protocol for resistance measurement, counts were compared using both sides of the fish and just one side. All lice were counted on a total of 550 fish—149 fish exposed for 7 days and 401 fish exposed for 17 days. On the remaining 855 fish, only the lice on the right side of the fish were counted.

At 17 dpi, the mean whole-body louse count was 54.5 ± 1.2 (mean ± s.e.m.). The head, body, tail fin, anal fin, pelvic fin, dorsal fin and pectoral fins were counted separately. The body contained more lice than any other region (14.3 ± 0.5) followed by the pectoral fin (13.0 ± 0.3). At 7 dpi, the mean louse count on the right side was 26.2 ± 0.5, compared to 27.0 ± 0.5 at 17 dpi. Mean louse count did not vary significantly from the chalimus I to the chalimus II stages (figure 1) suggesting that parasite loss was minimal between 7 and 17 dpi.

**Figure 3.**
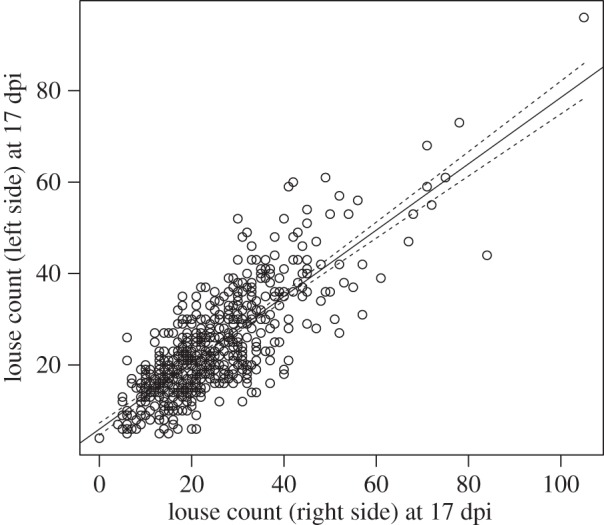
The correlation between louse counts obtained from left and right sides of the counted salmon. Lice were counted on both sides of 550 fish. There was a strong correlation (*R* = 0.8; *p* < 0.001), indicating that good estimates of parasite load may be obtained from one side of the fish, at reduced cost and effort. The dashed lines represent 95% confidence limits for mean predicted values of the linear regression line.

There was a strong correlation (*R* = 0.8; *p* < 0.001; figure 3) between lice counts from the left and right sides of the fish, indicating that good estimates of parasite load may be obtained from one side of the fish, at reduced cost and effort. The distribution of sea lice was right skewed (figure 2) with substantial variation between individuals: most fish have relatively low counts but a small number have high counts.

**Figure 4.**
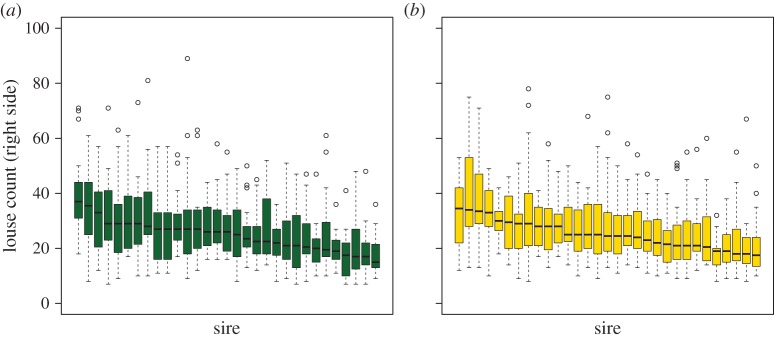
The median number of lice counted from 31 sire families at (*a*) 7 dpi and (*b*) 17 dpi, with families arranged in order of susceptibility from least susceptible to most susceptible. Box plots show the median (thick horizontal line), interquartile range (box), minimum and maximum values excluding outliers (whiskers) and outliers (circles) of the number of lice for each sire.

We found significant differences in size-corrected louse counts among families at both time points (figure 4*a*,*b*). Heritability estimates were not significantly different at 7 (0.27 ± 0.08) and 17 dpi (0.31 ± 0.08), giving a pooled estimate of 0.30 ± 0.06. These results indicate that differences among families are probably established early during the infection process and maintained through to the chalimus II stage.

### 3.2. Epidemiological model with selective breeding and sea lice control

**Figure 5.**
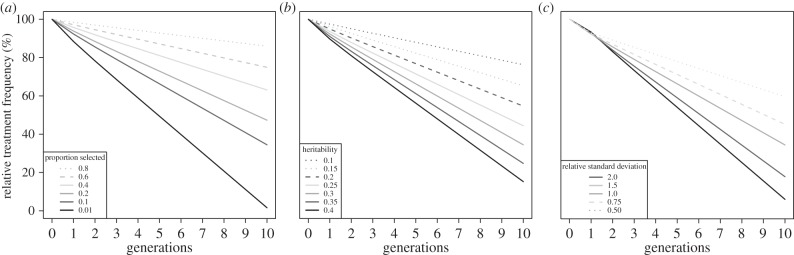
The modelled response to selection for reduced numbers of lice following infection in terms of required treatment frequency. (*a*) Sensitivity of response to differing proportions of the total population selected for breeding of the next generation. (*b*) Sensitivity of response to different heritabilities for a proportion selected of 0.1 and observed trait variance. (*c*) Sensitivity of response to different variance in susceptibility prior to selection for observed heritability of 0.3 and a proportion selected of 0.1.

We predicted the response to selection in terms of the reduction in mean parasite load across the fish population and in the required frequency of treatment over the 2 year production cycle, following Revie *et al*. [34]. Our model predicts clear reductions from generation 1 in the required frequency of drug treatment to maintain the same degree of parasite control as in the unselected population (figure 5*a*) although reductions occur much more rapidly for the higher intensities of selection. After five generations of selection, the required treatment frequency is reduced by about 5% for a selection intensity of 80% (i.e. most fish are retained for breeding) and by about 50% for a selection intensity of 1% (i.e. only the best 1% are used for breeding). Our default heritability from our experimental trials was 0.3. A heritability of 0.2 would slow the response to selection by nearly three generations, while a heritability of 0.4 would speed the response by nearly two generations (figure 5*b*). A reduction in the initial standard deviation in susceptibility to lice of 50% would slow the response by two to three generations, while a doubling of the trait standard deviation would speed the response by around four generations (figure 5*c*).

**Figure 6.**
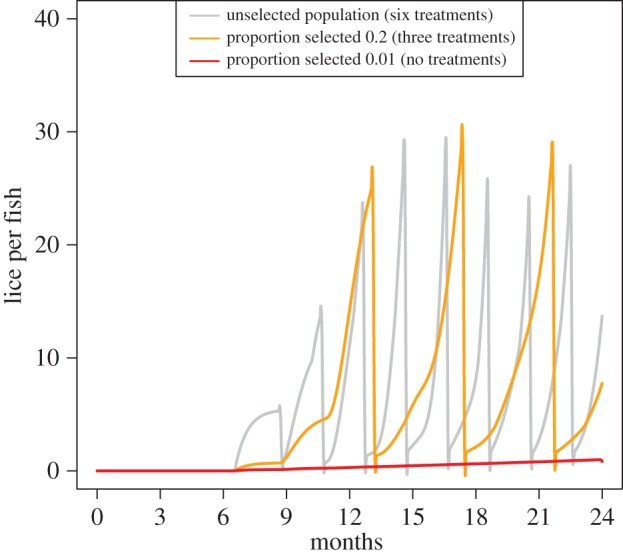
Predicted outbreaks following the use of three selection schemes and associated treatment regimes. In the unselected population (grey line), six treatments keep lice populations below 20–25 lice per fish; after 10 generations of selection on the best 0.2 of the population (orange lines), three treatments are sufficient to keep lice densities below this level, after 10 generations of selection on the best 0.01 of the populations (red line), treatments are not needed to control the lice population.

The need for treatment of lice population in unselected populations and populations following 10 generations of selection differed. Assuming drug efficacies for cypermethrin adopted by Revie *et al*. [34] (table 1), we found that in the unselected population, six treatments can keep lice populations below 20–25 lice per fish (figure 6, grey line); after 10 generations of selection on the best 0.2 of the population, three treatments are sufficient to keep lice densities below this level (figure 6, orange line); after 10 generations of selection on the best 0.01 of the populations, our model predicts that treatments are not needed to control the lice population (figure 6, red line).

## 4. Discussion

Selective breeding is a key element of disease management in intensive animal production and provides a cheap and effective alternative in situations where parasite resistance to chemical treatments is becoming a concern [36,37]. Selective breeding against a number of diseases is now used in aquaculture [38,39]. Although the potential for breeding salmon resistant to sea lice has been recognized [24–26,40,41], the industry has lacked reliable methods for estimating breeding values and the response to selection has not been assessed. Here, we provide a practical protocol for quantifying resistance to sea lice and show that selection could substantially reduce the need for drug treatments.

Counting multiple immature sea lice on large numbers of fish is a labour-intensive and demanding task. Therefore, we explored whether counting only one side of each fish would provide an accurate measure of host resistance. Counting lice on both sides of each fish would improve the precision with which the relative susceptibility of each animal can be estimated and generate a higher heritability (it would increase from 0.3 to 0.32), but the reduced effort required to count only one side of each fish would allow more fish to be counted and encourage adoption of the procedure by fish breeders. The analysis of sea lice counts following tank infection challenge has shown that there is a strong correlation between counts on the left- and right-hand sides of each fish. This means that it is possible to use counts from a single side to estimate susceptibility to infection.

The timing of sea lice counts following deliberate infection is important. Adult lice are larger and easier to count than the developmental stages, but their mobility means that counts of adult lice do not reflect the resistance status of the host. The number of lice was very similar at 7 and 17 dpi. These results indicate that the variation in resistance among young fish is a consequence of mechanisms acting against establishment and initial survival. There was little evidence for effective resistance against established chalimus stages. These results inform the mechanisms of immunity and also mean that sea lice counting can be flexible so long as it is carried out during the chalimus stages.

The distribution of lice was right skewed and overdispersed. A relatively small number of fish had high counts while most fish had relatively low counts. A skewed distribution of parasites is observed in the majority of parasitic infections [42–44]. This may be because some hosts are more susceptible than others or because infecting parasites are not evenly distributed in the infection environment. In tank infections the latter is less likely. The causes of differential susceptibility to infection are partly genetic [45,46] but the physiological mechanisms and the specific genes involved are not known. However, as mean lice counts remained unchanged from 7 to 17 dpi, this suggests that the physiological mechanisms were active before day 7. The swimming performance of infectious copepodids is not sufficient to follow fast-moving salmon, only to intercept them as they pass and therefore the mechanisms underlying variation are unlikely to involve the release of a chemical trail from fish, although there could feasibly be chemical differences at the surface of the fish. As the adaptive immune response takes more than 7 days to develop, the innate immune response against the incoming copepodids is likely to be the main factor determining the relative susceptibility of young fish. This could be a direct response or a consequence of the host's ability to evade any immunosuppressive factors secreted by the developing lice.

The ranking of families was very stable during the chalimus stages from 7 to 17 dpi. This suggests that, at the seawater temperature tested, these timings provide a convenient window to estimate variation in response to tank challenge infection. Prior to 7 days, sea lice are not permanently attached by filament and may become detached in handling and are, moreover, smaller and more difficult to find and count. Soon after 17 days, lice mature into pre-adults and are capable of moving from fish to fish. In this instance, relatively resistant fish, which prevented the development of larvae, could be infected subsequently by pre-adults and mistakenly appear susceptible. Overall, a relatively long window of stable counts of sessile lice simplifies the logistics of identifying resistant and susceptible fish.

The heritability of sea louse abundance was not significantly different at days 7 and 17; a pooled estimate was 0.30 with a standard error of 0.06, similar to or exceeding previous estimates obtained in Scottish and Norwegian salmon [24–26]. This is similar to the heritability of milk production in dairy cattle [29] and sufficiently high to justify selective breeding.

Our mathematical model allowed prediction of the response to selection. Based on our model, which has been fitted to field data from Scottish salmon farms [34] and parametrized using field trials to assess genetic variation in susceptibility, breeding for resistance to sea lice would reduce levels of infective larvae by reducing the number of individuals with high numbers of lice. This environmental benefit is not captured in traditional methods of estimating the response to selective breeding and was achieved by integrating quantitative genetics into an epidemiological model [47–49], allowing genetic variation between individuals and selective breeding to be modelled.

Selective breeding reduces the number of lice because hosts become more resistant and fewer lice transmission stages are produced. Consequently, the requirement for treatment is reduced. Such treatment reduction has the potential to prevent or slow the development of chemical resistance in treated populations, thus extending the life of developed medicines and improving longer term control [26,37,50]. In particular, the ability in salmon aquaculture to select a small number of sires and produce large numbers of offspring suggests that a relatively rapid response to selection is possible. Therefore, selective breeding offers a cheap and relatively rapid method that can form a key part of integrated pest management strategy for sea lice control.

In livestock, parasites contribute to many major diseases. Examples include the cattle tick *Rhipicephalus microplus* in Australian cattle [51,52] and nematodes in sheep across the world [45,53,54]. Additional problems are caused by flies, fleas, flukes and lice [55]. Disease susceptibility can also be influenced by many factors such as stress, nutrition, coinfection, intensity of exposure and parasite-mediated immunosuppression [56–58]. In estimating the heritability of disease, these factors influence the non-genetic component but they do not need to be explicitly captured. Here, the combination of experimental challenge, diagnostics, quantitative genetics and epidemiological modelling has provided a comprehensive framework for parasite control. This combination of disciplines could in principle be applied to develop control methods for a wide variety of diseases of managed populations of livestock and fish.

Sea lice currently pose a substantial problem for the aquaculture industry: they impact the welfare of farmed fish; threaten wild populations; and limit the profitability and future growth of the industry [59]. We have demonstrated through the integration of field trials, quantitative genetics and mathematical modelling, that selective breeding could substantially reduce the need for chemical treatments against sea lice. Selective breeding therefore offers the opportunity for more profitable, more ecologically sound and welfare-friendly fish farming.

## Data accessibility

The data obtained in this experiment are available by contacting M.S.

## References

[1] Frazer LN, Morton A, Krkosek M 2012 Critical thresholds in sea lice epidemics: evidence, sensitivity and subcritical estimation. Proc. R. Soc. B 279, 1950–1958. (10.1098/rspb.2011.2210) PMC3311887 22217721

[2] Costello MJ 2009 The global economic cost of sea lice to the salmonid farming industry. J. Fish Dis. 32, 115–118. (10.1111/j.1365-2761.2008.01011.x) 19245636

[3] Oelckers K, Vike S, Duesund H, Gonzalez J, Wadsworth S, Nylund A 2014 *Caligus rogercresseyi* as a potential vector for transmission of Infectious Salmon Anaemia (ISA) virus in Chile. Aquaculture 420, 126–132. (10.1016/j.aquaculture.2013.10.016)

[4] Burridge LE, Weis JS, Cabello F, Pizarro J, Bostick K 2010 Chemical use in salmon aquaculture: a review of current practices and possible environmental effects. Aquaculture 306, 7–23. (10.1016/j.aquaculture.2010.05.020)

[5] Langford KH, Øxnevad S, Schøyen M, Thomas KV 2014 Do antiparasitic medicines used in aquaculture pose a risk to the Norwegian Aquatic Environment? Environ. Sci. Technol. 48, 7774–7780. (10.1021/es5005329) 24905382

[6] Burridge LE, Lyons MC, Wong DKH, MacKeigan K, VanGeest JL 2014 The acute lethality of three anti-sea lice formulations: AlphaMax®, Salmosan®, and Interox®Paramove™50 to lobster and shrimp. Aquaculture 420–421, 180–186. (10.1016/j.aquaculture.2013.10.041)

[7] Van Geest JL, Burridge LE, Kidd KA 2014 The toxicity of the anti-sea lice pesticide AlphaMax® to the polychaete worm *Nereis virens*. Aquaculture 430, 98–106. (10.1016/j.aquaculture.2014.03.044)

[8] Costello MJ 2006 Ecology of sea lice parasitic on farmed and wild fish. Trends Parasitol. 22, 475–483. (10.1016/j.pt.2006.08.006) 16920027

[9] Torrissen O, Jones S, Asche F, Guttormsen A, Skilbrei OT, Nilsen F, Horsberg TE, Jackson D 2013 Salmon lice—impact on wild salmonids and salmon aquaculture. J. Fish Dis. 36, 171–194. (10.1111/jfd.12061) 23311858 PMC3675643

[10] Krkosek M, Connors BM, Morton A, Lewis MA, Dill LM, Hilborn R 2011 Effects of parasites from salmon farms on productivity of wild salmon. Proc. Natl Acad. Sci. USA 108, 14 700–14 704. (10.1073/pnas.1101845108) PMC3167527 21873246

[11] Krkosek M, Ford JS, Morton A, Lele S, Myers RA, Lewis MA 2007 Declining wild salmon populations in relation to parasites from farm salmon. Science 318, 1769–1772. (10.1126/science.1148744) 18079401

[12] Rees EE, St-Hilaire S, Jones SRM, Krkosek M, DeDominicis S, Foreman MGG, Patanasatienkul T, Revie CW 2015 Spatial patterns of sea lice infection among wild and captive salmon in western Canada. Landsc. Ecol. Springer Netherlands 30, 989–1004. (10.1007/s10980-015-0188-2)

[13] Marty GD, Saksida SM, Quinn TJ 2010 Relationship of farm salmon, sea lice, and wild salmon populations. Proc. Natl Acad. Sci. USA 30, 989–1004. (10.1073/pnas.1009573108) PMC3012511 21149706

[14] Lees F, Baillie M, Gettinby G, Revie CW 2008 The Efficacy of Emamectin Benzoate against Infestations of *Lepeophtheirus salmonis* on Farmed Atlantic Salmon (*Salmo salar* L) in Scotland, 2002–2006. PLoS ONE 3, e1549 (10.1371/journal.pone.0001549) 18253496 PMC2212131

[15] Jones PG, Hammell KL, Gettinby G, Revie CW 2013 Detection of emamectin benzoate tolerance emergence in different life stages of sea lice, *Lepeophtheirus salmonis*, on farmed Atlantic salmon, *Salmo salar* L. J. Fish Dis. 36, 209–220. (10.1111/jfd.12022) 23347188

[16] Aaen SM, Helgesen KO, Bakke MJ, Kaur K, Horsberg TE 2015 Drug resistance in sea lice: a threat to salmonid aquaculture. Trends Parasitol. 31, 72–81. (10.1016/j.pt.2014.12.006) 25639521

[17] Igboeli OO, Burka JF, Fast MD 2013 *Lepeophtheirus salmonis*: a persisting challenge for salmon aquaculture. Anim. Front. 4, 22–32. (10.2527/af.2014-0004)

[18] McNair CM 2015 Ectoparasites of medical and veterinary importance: drug resistance and the need for alternative control methods. J. Pharm. Pharmacol. 67, 351–363. (10.1111/jphp.12368) 25644683

[19] Liu Y, Bjelland HV 2014 Estimating costs of sea lice control strategy in Norway. Prev. Vet. Med. 117, 469–477. (10.1016/j.prevetmed.2014.08.018) 25443395

[20] Brooks KM 2009 Considerations in developing an integrated pest management programme for control of sea lice on farmed salmon in Pacific Canada. J. Fish Dis. 32, 59–73. (10.1111/j.1365-2761.2008.01013.x) 19245631

[21] Nicholas FW 2005 Animal breeding and disease. Phil. Trans. R. Soc. B 360, 1529–1536. (10.1098/rstb.2005.1674) 16048793 PMC1569506

[22] Moen T 2010 Breeding for disease resistance to viral diseases in salmonids. In Breeding for disease resistance in farm animals (eds Bishop SC, Axford RFE, Nicholas FW, Owen JB), pp. 166–179, 3rd edn Wallingford: CABI.

[23] Stear MJ 2010 Breeding for resistance to nematodes. In Breeding for disease resistance in farm animals (eds Bishop SC, Axford RFE, Nicholas FW, Owen JB), pp. 166–179, 3rd edn Wallingford, UK: CABI.

[24] Glover KA, Aasmundstad T, Nilsen F, Storset A, Skaala Ø 2005 Variation of Atlantic salmon families (*Salmo salar* L.) in susceptibility to the sea lice *Lepeophtheirus salmonis* and *Caligus elongatus*. Aquaculture 245, 19–30. (10.1016/j.aquaculture.2004.11.047)

[25] Kolstad K, Heuch P, Gjerde B, Gjedrem T, Salte R 2005 Genetic variation in resistance of Atlantic salmon (*Salmo salar*) to the salmon louse *Lepeophtheirus salmonis*. Aquaculture 247, 145–151. (10.1016/j.aquaculture.2005.02.009)

[26] Gjerde B, Ødegård J, Thorland I 2011 Estimates of genetic variation in the susceptibility of Atlantic salmon (*Salmo salar*) to the salmon louse *Lepeophtheirus salmonis*. Aquaculture 314, 66–72. (10.1016/j.aquaculture.2011.01.026)

[27] Falconer DS, MacKay TFC 1996 Introduction to quantitative genetics, 4th edn Harlow, UK: Pearson Education Limited.

[28] Hamre LA, Eichner C, Caipang CMA, Dalvin ST, Bron JE, Nilsen F, Boxshall G, Skern-Mauritzen R 2013 The salmon louse *Lepeophtheirus salmonis* (Copepoda: Caligidae) life cycle has only two chalimus stages. PLoS ONE 8, e73539 (10.1371/journal.pone.0073539) 24069203 PMC3772071

[29] Nicholas FW 2010 Introduction to veterinary genetics, 3rd edn Chichester, UK: Wiley-Blackwell.

[30] Lynch M, Walsh B 1988 Genetics and analysis of quantitative traits. Sunderland, MA: Sinauer Associates Inc.

[31] R Core Team. 2015 R: a language and environment for statistical computing. Vienna, Austria: R Foundation for Statistical Computing.

[32] Gilmour AR, Gogel BJ, Cullis BR, Welham SJ, Thompson R 2002 ASReml User Guide Release 1.0, 2nd edn UK: NSW Agriculture Biometrical Bulletin. Hemel Hempstead.

[33] Wilson AJ, Réale D, Clements MN, Morrissey MM, Postma E, Walling CA, Kruuk LEB, Nussey DH 2010 An ecologist's guide to the animal model. The J. Anim. Ecol. 79, 13–26. (10.1111/j.1365-2656.2009.01639.x) 20409158

[34] Revie CW, Robbins C, Gettinby G, Kelly L, Treasurer JW 2005 A mathematical model of the growth of sea lice, *Lepeophtheirus salmonis*, populations on farmed Atlantic salmon, *Salmo salar* L., in Scotland and its use in the assessment of treatment strategies. J. Fish Dis. 28, 603–613. (10.1111/j.1365-2761.2005.00665.x) 16302954

[35] Bulmer MG 1971 The effect of selection on genetic variability. Am. Nat. 105, 201–211. (10.1086/282718)

[36] Stear MJ, Bishop SC, Mallard BA, Raadsma H 2001 The sustainability, feasibility and desirability of breeding livestock for disease resistance. Res. Vet. Sci. 71, 1–7. (10.1053/rvsc.2001.0496) 11666141

[37] Stear MJ, Nikbakht G, Matthews L, Jonsson NN 2012 Breeding for disease resistance in livestock and fish. CAB Rev. Perspect. Agric. Vet. Sci. Nutr. Nat. Resour. 7, 1–10. (10.1079/PAVSNNR20127007)

[38] Henryon M, Berg P, Olesen NJ, Kjær TE, Slierendrecht WJ, Jokumsen A, Lund I 2005 Selective breeding provides an approach to increase resistance of rainbow trout (*Onchorhynchus mykiss*) to the diseases, enteric redmouth disease, rainbow trout fry syndrome, and viral haemorrhagic septicaemia. Aquaculture 250, 621–636. (10.1016/j.aquaculture.2004.12.022)

[39] Bishop SC, Axford RFE, Nicholas FW, Owen JB (eds). 2010 Breeding for disease resistance in farm animals, 3rd edn Wallingford, CT: CABI.

[40] Mustafa A, MacKinnon BM 1999 Genetic variation in susceptibility of Atlantic salmon to the sea louse *Caligus elongatus* Nordmann, 1832. Can. J. Zool. 77, 1332–1335. (10.1139/cjz-77-8-1332)

[41] Jones CS, Lockyer AE, Verspoor E, Secombes CJ, Noble LR 2002 Towards selective breeding of Atlantic salmon for sea louse resistance: approaches to identify trait markers. Pest Manage. Sci. 58, 559–568. (10.1002/ps.511) 12138622

[42] Gaba S, Ginot V, Cabaret J 2005 Modelling macroparasite aggregation using a nematode-sheep system: the Weibull distribution as an alternative to the Negative Binomial distribution? Parasitology 131, 393–401. (10.1017/S003118200500764X) 16178361

[43] Wilson K, Grenfell BT, Shaw DJ 1996 Analysis of aggregated parasite distributions: a comparison of methods. Funct. Ecol. 10, 592–601. (10.2307/2390169)

[44] Anderson RM, Gordon DM 1982 Processes influencing the distribution of parasite numbers within host populations with special emphasis on parasite-induced host mortalities. Parasitology 85, 373–398. (10.1017/S0031182000055347) 7145478

[45] Stear MJ, Boag B, Cattadori I, Murphy L 2009 Genetic variation in resistance to mixed, predominantly *Teladorsagia circumcincta* nematode infections of sheep: from heritabilities to gene identification. Parasite Immunol. 31, 274–282. (10.1111/j.1365-3024.2009.01105.x) 19388948

[46] Stear MJ, Wakelin D 1998 Genetic resistance to parasitic infection. Revue scientifique et technique (International Office of Epizootics) 17, 143–153. 9638807 10.20506/rst.17.1.1089

[47] Bishop SC, Stear MJ 1997 Modelling responses to selection for resistance to gastro-intestinal parasites in sheep. Anim. Sci. 64, 469–478. (10.1017/S1357729800016088)

[48] MacKenzie KM, Bishop SC 2001 Utilizing stochastic genetic epidemiological models to quantify the impact of selection for resistance to infectious diseases in domestic livestock. J. Anim. Sci. 79, 2057–2065. 11518213 10.2527/2001.7982057x

[49] Prada Jimenez de Cisneros J, Stear MJ, Mair C, Singleton D, Stefan T, Stear A, Marion G, Matthews L 2014 An explicit immunogenetic model of gastrointestinal nematode infection in sheep. J. R. Soc. Interface 11, 20140416 (10.1098/rsif.2014.0416) 25121649 PMC4233724

[50] Jackson F, Miller J 2006 Alternative approaches to control—Quo vadit? Vet. Parasitol. 139, 371–384. (10.1016/j.vetpar.2006.04.025) 16750600

[51] Stear MJ, Hetzel DJ, Brown SC, Gershwin LJ, Mackinnon MJ, Nicholas FW 1990 The relationships among ecto- and endoparasite levels, class I antigens of the bovine major histocompatibility system, immunoglobulin E levels and weight gain. Vet. Parasitol. 134, 303–321. (10.1016/0304-4017(90)90077-O) 2316176

[52] Jonsson NN 2006 The productivity effects of cattle tick (*Boophilus microplus*) infestation on cattle, with particular reference to *Bos indicus* cattle and their crosses. Vet. Parasitol. 137, 1–10. (10.1016/j.vetpar.2006.01.010) 16472920

[53] Stear MJ, Murray M 1994 Genetic resistance to parasitic disease: particularly of resistance in ruminants to gastrointestinal nematodes. Vet. Parasitol. 54, 161–176. (10.1016/0304-4017(94)90089-2) 7846849

[54] Morris CA, Wheeler M, Watson TG, Hosking BC, Leathwick DM 2005 Direct and correlated responses to selection for high or low faecal nematode egg count in Perendale sheep. N Z J. Agric. Res. 48, 1–10. (10.1080/00288233.2005.9513625)

[55] Taylor MA, Coop RL, Wall R 2007 Veterinary parasitology, 3rd edn Oxford, UK: Wiley-Blackwell.

[56] MacKinnon BM 1998 Host factors important in sea lice infections. ICES J. Mar. Sci. 55, 188–192. (10.1006/jmsc.1997.0361)

[57] Coop RL, Kyriazakis I 2001 Influence of host nutrition on the development and consequences of nematode parasitism in ruminants. Trends Parasitol. 17, 325–330. (10.1016/S1471-4922(01)01900-6) 11423375

[58] Stear MJ, Bishop SC, Henderson NG, Scott I 2003 A key mechanism of pathogenesis in sheep infected with the nematode *Teladorsagia circumcincta*. Anim. Health Res. Rev. 4, 45–52. (10.1079/AHRR200351) 12885208

[59] Jansen PA, Kristoffersen AB, Viljugrein H, Jimenez D, Aldrin M, Stien A 2012 Sea lice as a density-dependent constraint to salmonid farming. Proc. R. Soc. B 279, 2330–2338. (10.1098/rspb.2012.0084) PMC3350688 22319130

